# Breast Cancer Stem Cells: Biomarkers, Identification and Isolation Methods, Regulating Mechanisms, Cellular Origin, and Beyond

**DOI:** 10.3390/cancers12123765

**Published:** 2020-12-14

**Authors:** Xiaoli Zhang, Kimerly Powell, Lang Li

**Affiliations:** Department of Biomedical Informatics, College of Medicine, The Ohio State University, 320B Lincoln Tower, 1800 Cannon Dr., Columbus, OH 43210, USA; kimerly.powell@osumc.edu

**Keywords:** breast cancer, stem cells, biomarkers, identification and isolation, mechanism, cellular origin, lineage tracing

## Abstract

**Simple Summary:**

Breast cancer stem cells are blamed to be responsible for breast cancer tumorigenesis, metastasis, drug resistance and tumor recurrence. Therefore, it is critical to identify this subset of cells and understand their molecular mechanisms for the development of breast cancer treatment strategies. Here, we review the recent advances in breast cancer stem cell studies in terms of available biomarkers, identification and isolation methods, molecular mechanisms, and methods for studying their cellular origin and lineage development.

**Abstract:**

Despite recent advances in diagnosis and treatment, breast cancer (BC) is still a major cause of cancer-related mortality in women. Breast cancer stem cells (BCSCs) are a small but significant subpopulation of heterogeneous breast cancer cells demonstrating strong self-renewal and proliferation properties. Accumulating evidence has proved that BCSCs are the driving force behind BC tumor initiation, progression, metastasis, drug resistance, and recurrence. As a heterogeneous disease, BC contains a full spectrum of different BC subtypes, and different subtypes of BC further exhibit distinct subtypes and proportions of BCSCs, which correspond to different treatment responses and disease-specific outcomes. This review summarized the current knowledge of BCSC biomarkers and their clinical relevance, the methods for the identification and isolation of BCSCs, and the mechanisms regulating BCSCs. We also discussed the cellular origin of BCSCs and the current advances in single-cell lineage tracing and transcriptomics and their potential in identifying the origin and lineage development of BCSCs.

## 1. Introduction

Breast cancer (BC) is one of the most common leading causes of cancer-related death in women worldwide [1]. Despite the recent advances in diagnosis and treatment strategies, patients under remission may still develop relapse and metastasis, which is a major cause of mortality among BC patients [2]. BC is considered a heterogeneous disease with a spectrum of many different subtypes and stages that lead to different treatment responses and disease-specific outcomes [3,4]. Different subtypes of BC can be identified primarily with immunohistochemistry (IHC) [5] and gene expression profiling [6]. According to the IHC/fluorescence in situ hybridization (FISH) profile, BC can be classified and divided on the basis of presence of the estrogen receptor (ER), the progesterone receptor (PR), and human epidermal growth factor receptor 2 (HER2) into ER-positive, HER2-positive, and triple-negative BC (TNBC) that is defined by the absence of ER, PR, and HER2 [4]. Among the three immunohistochemical subtypes of BC, TNBC, representing ~20% of all BC cases, is associated the most with poor prognosis and worse survival due to early metastasis to other organs and a lack of clinically established targeted therapies [7,8,9,10]. At the molecular level, gene expression profiling has defined five major subtypes of BC: luminal A, luminal B, HER2-enriched, basal, and normal-like [6,9,11,12,13]. TNBC forms the largest part of the basal-like subtype (~80%) [6,14], which is the most aggressive molecular subtype with the highest content of breast cancer stem cells (BSCSs) characterized by the most common BCSC biomarkers, CD44^+^/CD24^−/low^ and ALDH1^+^ [15,16,17,18].

Accumulating evidence has demonstrated that cancer stem cells (CSCs) are the driving force leading to BC tumor progression, metastasis, and resistance to conventional therapy [15,19,20,21,22]. CSCs, also called tumor-initiating cells (TICs), accounting for only 0.1–1% of all tumor cells, is a small but significant subpopulation of undifferentiated cells in tumors [23]. This subpopulation of cells is capable of self-renewal and differentiation into all the different cell types that cause tumor formation and subsequent metastasis [24]. In addition, recent studies demonstrated that the number of cells with tumorigenic potential, i.e., of the CSCs, determines tumor heterogeneity [25,26,27].

**Figure 1:**
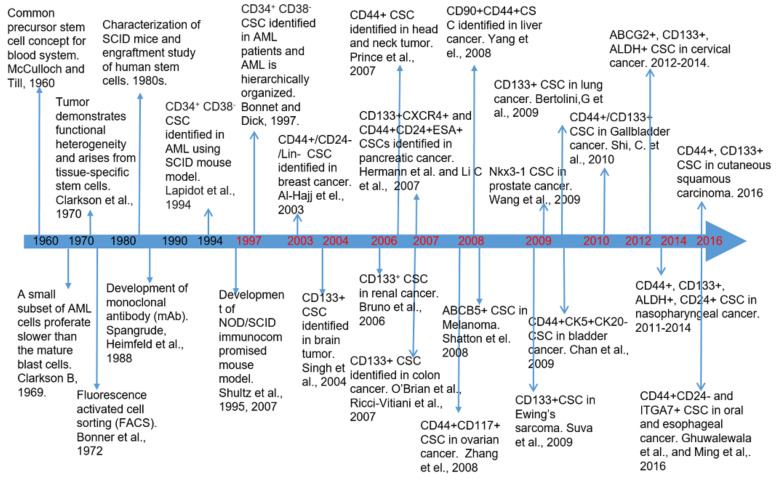
Timeline of the important milestones before cancer stem cells were first identified in AML in 1997 (years marked in black) and CSC identification in different cancer types (years marked in red) since then to date.

The concept of CSCs dates back to 1937 when Furth and Kahn demonstrated that a single murine leukemia cell could initiate a tumor in mice [28]. However, in the following years, works showed a wide variation of tumor initiation frequency, especially the studies with human tumor cells showing that the tumor-initiating cells are rare and the required number of such cells to form a tumor is higher than 10^6^ [29]. Starting from 1960s, several studies, including the demonstration of a common precursor stem cell for cells in the blood system [30], the concept of tumor functional heterogeneity [31], and the identification of a small subset of cells proliferating slower than the mature blast cells in acute myeloid leukemia (AML) [32], along with the development of monoclonal antibodies (mAbs) [33] and fluorescence-activated cell sorting (FACS) [34], laid the foundation for the seminal discovery of AML stem cells with the CD38^+^/CD34^−^ phenotype in a mouse model by John Dick and colleagues in 1994 [29,35] (Figure 1). Based on these observations and techniques, further enhanced by the development of a NOD/SCID (non-obese diabetic/severe combined immune deficiency) mouse model [36] for the xenotransplantation assay, the first identification of CSCs in AML became possible in 1997 [37]. That study showed that the human AML initiated with a very few FAC-sorted CD34^+^/CD38^−^ AML cells is similar to normal hematopoietic stem cells and can be serially passaged in NOD/SCID mice [37]. Following this study, the identification of CSCs in BC with the CD44^+^/CD24^−/low^ phenotype started the application of these technologies and observations to solid cancers [38]. The first decade of this century has evidenced an avalanche of reports on the identification of CSCs in most solid tumors [39,40]. In the following years, the CSCs with different surface markers were subsequently identified in brain cancer [41], colon cancer [42,43,44], head and neck cancer [45], pancreatic cancer [46,47], melanoma [48], liver cancer [49], ovarian cancer [50], lung cancer [51], prostate cancer [52], bladder cancer [53], Ewing’s sarcoma [54], and several other cancers [40,55] (Figure 1). These CSCs possess specific surface markers, such as CD133^+^, CD44^+^, CD24^−^, CD34^+^, CD29^+^, CD38^−^, CD166^+^, epithelial cell adhesion molecule (EpCAM), and Lin^−^, that enable these cells to be isolated with FACS or other immunoselection procedures [40,56].

In this review, we aimed to discuss the current knowledge on BCSC biomarkers and their relative abundance and clinical relevance in different subtypes of BC, methods for identification and isolation of BCSCs, the mechanism regulating BCSCs and their therapeutic potential, theories of BCSC origin, and methods for studying BCSC lineage development with the combination of the novel technology of single-cell lineage tracing and transcriptomics.

## 2. Biomarkers of Breast Cancer Stem Cells and Their Clinical Relevance

Since BCSCs were first identified in 2003 based on CD44 and CD24 expression [38], different biomarkers for BCSCs have been identified in BC patient tumor samples, animal models, and cell lines, indicating the existence of a variety of BCSC subgroups. Furthermore, different BC subtypes demonstrate variation in the proportion of different BCSC subgroups, which corresponds to different patient treatment responses and clinical outcomes.

### 2.1. Breast Cancer Stem Cell Biomarkers

**Figure 2:**
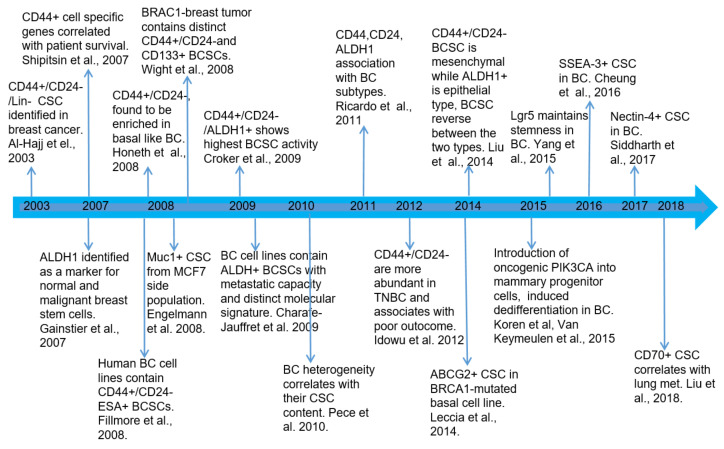
Timeline of important discoveries and findings of breast cancer stem cell studies since the cancer stem cells were initially identified in breast cancer in 2003.

Al-Hajj and colleagues were the first to identify tumor-initiating CSCs in BC by using cell surface markers CD44 and CD24 [38]. They showed that a small subpopulation of cells (as few as 100 cells) with the CD44^+^/CD24^−^/lin^−^ phenotype was able to produce tumors with similar heterogeneity to that of the original tumor in immunodeficient mice, while other tumor cells, even with as many as 10^5^ to 10^6^ cells, were unable to produce tumors in mice of the same type. In addition, the single cell suspensions of CD44^+^/CD24^−^/lin^−^ cells from human BC cells were able to self-renew, proliferate extensively, form clonal mammospheres (a property of cancer stemness), and display chemotherapy resistance in an in vitro cell culture system [57,58]. Later, aldehyde dehyogenase 1 (ADLH1) was identified as a marker of both normal and malignant human mammary stem cells and a predictor of poor patient outcomes [16]. Additional markers for characterizing BCSCs, such as ABCG2, CD133, CD49f, LGR5, SSEA-3, CD70, and PROCR, have been recently reported [59,60,61,62,63,64,65,66]. Figure 2 shows the timeline of important discoveries and findings during the BCSC studies. The identification of BCSC populations is not restricted to patient tumors or primary cells, but they were also identified in established BC cell lines with different cell lines showing various proportions of different BCSC phenotypes [58,67].

To date, the most consistently used biomarkers for the identification of BCSC phenotypes are CD44, CD24, and ALDH1 [68]. Accumulating evidence has shown that BCSCs with ALDH1^+^ and the CD44^+^/CD24^−/low^ phenotype are responsible for tumor initiation, progression, metastasis, and drug resistance [67,69,70,71,72,73]. CD44 is a cell surface transmembrane glycoprotein that is involved in many cellular functions, including cellular adhesion, proliferation, survival, and differentiation. The elevated expression of CD44 in BCSCs acts to maintain the multipotency of the BCSC population [74]. CD24 is a sialoprotein that enhances cellular adhesion, proliferation, and metastasis [75]. While low or absence of CD24 expression is one of the features of BCSCs, arising of CD24^+^ cell populations was reported from radiation-treated CD24^−/low^ cells indicating a role of this protein in radio- and chemoresistance in breast cancer cell lines [76]. ALDH1 is a member of the aldehyde dehydrogenase family of proteins and can act as a modulator of several cell functions, including stem cell proliferation and differentiation [77]. The other less frequently used biomarkers involved in the identification of BCSCs include CD133, CD49f, CD61, PCOR [2], and CD90 [78]. CD133, also known as prominin-1, is a cell surface glycoprotein that was found in TNBC and BRCA-1-deficient mouse tumors [61,79]. Overexpression of CD133 is associated with a poor prognosis in patients with invasive BC [80]. CD49f and CD61 were found to be associated with BC tumor initiation properties in mice [81,82]. PROCR was identified based on gene expression profiling of primary BC tumors [60] and was later found primarily expressed in basal subpopulations [62]. It was demonstrated that CD90 is induced by the epithelial–mesenchymal transition (EMT) and the CD90^+^ population in TNBC contains BCSCs [78]. Some additional BCSC markers, such as MUC1, GD2, ABCG2, Lgr5, Nectin-4, and CD70, were identified in BC cell lines [83].

### 2.2. BCSC Subpopulations Are Heterogeneous with Different Subtypes

Detailed analysis revealed that CD44^+^/CD24^−/low^ and ALDH1 biomarkers identified largely nonoverlapping cell populations in primary human breast cancers [15,84]. BCSC subpopulations with CD44^+^/CD24^−/low^ markers display a mesenchymal and quiescent phenotype resembling those of basal stem cells that are more invasive, while BCSCs with the ALDH1^+^ phenotype show an epithelial and proliferative phenotype resembling those of luminal stem cells that are more localized [84]. While mesenchymal and epithelial are the two states during EMT or mesenchymal-to-epithelial transition (MET), BCSCs can reversibly transit between these two states under the regulation of cytokine signaling [84,85]. However, despite the significant differences between cells characterized by ALDH1^+^ and CD44^+^/CD24^−/low^ expression, both of these BCSC populations shared characteristics of stemness with being able to recreate a tumor in a xenograft model, and both displayed a remarkable similarity in gene expression patterns across the molecular subtypes of BC [84]. However, the study showed that neither CD44^+^/CD24^−/low^ nor ALDH1^+^ BCSCs show 100% sphere formation abilities in vitro [16]. The small populations of BCSCs that simultaneously express both ALDH1^+^ and CD44^+^/CD24^−/low^ biomarkers show the highest tumorigenic and metastatic activity; they were able to generate tumors with as few as 20 cells [16,86].

### 2.3. Relative Breast Cancer Stem Cell Abundance in Different Breast Cancer Subtypes and Their Clinical Relevance

The proportion of the CD44^+^/CD24^−/low^ BCSC subpopulations were found to differ in breast cancer subtypes, where basal-like tumors show a higher proportion of these cells than luminal type tumors [15,18]. Similarly, in cell lines, basal/mesenchymal BC cell lines are enriched in the CD44^+^/CD24^−/low^ phenotype, while luminal cell lines are enriched in the CD44^−/low^/CD24^+^ cell population, and basal/epithelial cell lines are enriched in CD44^+^/CD24^+^ cell populations [15]. Recent studies demonstrated that the CD44^+^/CD24^−/low^ phenotype is associated with poor prognosis of TNBC and metastatic BC patients [87,88,89,90]. However, opposite results where the CD44^+^/CD24^−/low^ phenotype was associated with favorable prognosis were reported by Kim et al. in a separate cohort study [91]. These contradictory results could be due to the different patient cohorts (such as patients of different ethnicity or different races) or BC subtypes used in different studies. Therefore, further studies with larger BC patient cohorts including different BC subtypes and/or ethnicity/races are needed to confirm the clinical relevance of the CD44^+^/CD24^−/low^ phenotype in predicting BC patient prognosis.

Elevated expression of ALDH1 identifies a subpopulation of BCSCs and correlates with poor prognosis of patients with TNBCs [16,92]. ALDH (aldehyde hydrogenase) activity is considered a better predictive marker for BCSCs as cells with high ALDH activity have a higher tumorigenic activity in vivo in comparison with cells having the CD44^+^/CD24^−/low^ phenotype [16]. Similar to CD44^+^/CD24^−/low^ cells, ALDH1^+^ BCSCs are more frequently found in basal-like breast cancer tumors than in luminal type tumors and cell lines; however, ALDH1^+^ cells are also commonly found in the HER2+ BC subtype [15,16].

Correspondingly, different subtypes of BC exhibit various abundance of BCSCs and varying proportions of epithelial or mesenchymal BCSC subtypes. TNBC contains the highest proportion of BCSCs, shows an increased degree of sphere formation [15,18,58,70,87,93,94], and is significantly enriched with stem cell gene signatures, such as c-KIT, TGF-β, α6-integrin subunit, and prion protein, compared to other non-TNBC cells [95,96], which contributes to the poor prognosis associated with this subtype [94,97]. TNBCs are enriched with both CD44^+^/CD24^−/low^ and ALDH1^+^ types of BCSCs, while claudin-low TNBCs are characterized by a higher proportion of mesenchymal BCSCs with CD44^+^/CD24^−/low^ expression, and basal-like TNBCs contain a higher proportion of ALDH1^+^ epithelial BCSCs and a subcomponent of mesenchymal BCSCs [98]. HER2+ BC is characterized by a high proportion of ALDH1^+^ epithelial BCSCs, but at a lower proportion than TNBC, followed by luminal B BC subtype displaying a certain proportion of BCSCs and luminal A BC exhibiting the lowest proportion of BCSCs [98]. HER2 strongly regulates the genes related to stem cell and progenitor cell control [99] and is selectively expressed in HER2−/ER+ luminal BCSCs [100]. The HER2+ CD44^+^/CD24^−/low^ CSCs isolated from HER2− BC cells showed enhanced ALDH activity and aggressiveness compared to those isolated from HER2+ BC [101]. Further evidence showed that HER2-low population sorted from the mammospheres of luminal A subtype MCF7 cells had increased stem cell properties and markers, such as OCT4, NANOG, and SOX2, as compared to those of the HER2-high population sorted from the same subtype of MCF7 cells [102].

## 3. Methods for the Identification and Isolation of Breast Cancer Stem Cells

**Figure 3:**
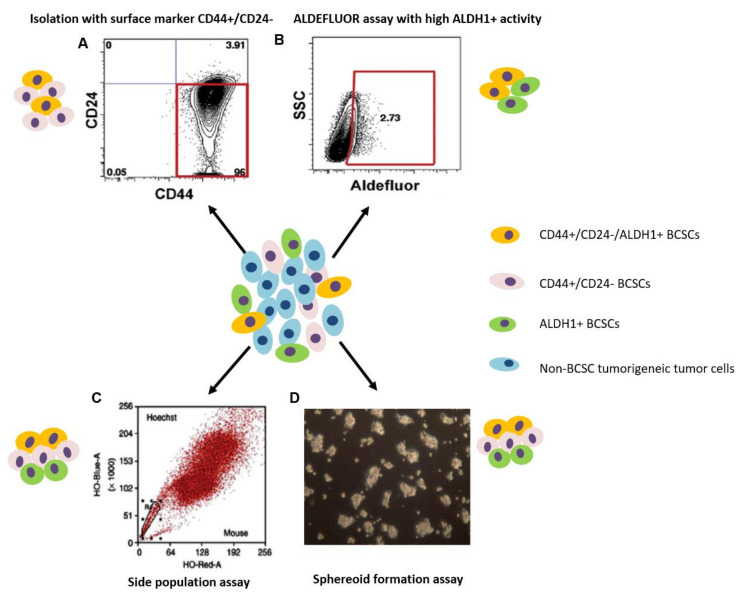
Major BCSC identification and isolation methods: isolation based on (**A**) BCSC surface markers CD44^+^CD24^−^, (**B**) ALDEFLUOR assay based on high ALDH1 activity, (**C**) side population assay based on high ABC transporter expression, and (**D**) spheroid formation assay based on the CSC capability of forming spheres. Cells with different colors to depict the heterogeneity of BC tumors and BCSC subtypes. The isolated BCSCs from all four methods have the capability to form heterogeneous tumors in vivo. SSC: side-scattered light. This figure is modified based on previous reviews, [56,29].

Currently, CSCs can be identified and isolated by four main methodologies that depend either on the high expression of cell surface markers or on the functional aspects of CSCs. The most commonly used method of CSC isolation is based on specific cell surface biomarkers or biomarker combinations, which is also a priority in cancer research. The other three commonly used methods, i.e., side population (SP) cell isolation with Hoechst 33342, ALDEFLUOR assay, and mammosphere formation, are mainly based on CSC intrinsic properties, including high expression of ATP-binding cassette (ABC) transporters, high expression of ALDH1, self-renewal and proliferation of CSCs, respectively [56] (Figure 3). These methods have been widely applied to isolate CSCs from cancer cell lines and different tumor tissues, including breast cancer.

### 3.1. Isolation with Cell Surface Markers

Using surface markers to isolate CSCs has become the most commonly used method for the isolation of CSCs and has been applied to isolate CSCs from heterogeneous tumor cell populations with different malignancies [56]. To identify and isolate CSCs, the selection of appropriate specific cell surface markers is the first priority and critical for a successful isolation. Fluorescence-activated cell sorting (FACS) or magnetic cell sorting (MACS) allows CSCs to be labeled with surface markers, sorted, and further tested/manipulated in the laboratory setting. The first evidence of the existence of CSCs was derived from AML using FACS based on the expression of cell surface markers CD34 and CD38 (CD34^+^CD38^−^) [37]. Since then, the CSCs have been isolated using FACS or MACS from many types of solid tumors, including breast cancer. The specific surface markers commonly used for isolation include but are not limited to CD24, CD44, CD133, CD13, CD14, CD15, CXCR4, EpCAM, LGR5, CD49f, CD90, CD117, etc. [103]. In breast cancer, CD24, CD44, CD133, EpCAM, CD49f, CD90, and CD61 are the commonly used markers, alone or in combination, for the identification and isolation of BCSCs, where the combination of biomarkers CD44^+^/CD24^−/low^/ESA^+^/Lin^−^ was used in the initial BCSC study (Figure 3) [38]. Later, the rest of the above-mentioned BC surface markers were also proved suitable for the identification of BCSCs in several in vivo and in vitro studies [78,79,81,82].

To isolate cells using FACS, the immune-stained cells will be sorted using fluorescently labeled antibodies targeting the selected surface markers. The FACS method allows cells in the cell suspension to pass through the flow cytometer as a narrow stream and to be separated based on the recognition of cell size, granularity, and fluorescent properties of individual cells by the laser detector [56]. In contrast, MACS allows the isolation and enrichment of stem cells without any staining [56]. This method separates cells based on whether cells are conjugated to magnetic nanoparticles using specific antibodies. Labeled cells will be transmitted into a column placed in a strong magnetic field, where cells expressing the antigen will bind to the magnetic beads via the antibodies and remain in the column, while all other cells not expressing the antigen will come off the column and be washed away [56]. Compared to FACS, which is a multiparametric method, the MACS method is simpler without the staining step and requires less complicated equipment, but it cannot isolate cells with multiple markers simultaneously, and therefore the efficacy is less satisfactory [56]. The detailed experimental steps for isolation of BCSCs based on cell surface markers can be found in the molecular biology protocol by Jia X et al. [104].

The advantage of isolation with cellular markers is that CSCs are more specific than those isolated with functional assays as described below. However, there are some limitations to this method. First, many of the surface markers for identification of CSCs are initially used for the identification of normal stem cells, such as embryonic stem cells and adult stem cells, which may cause concerns about the specificity and consistency of these markers [56]. Second, the lengthy complex procedure used for CSC isolation based on markers may cause possible damage of surface markers during sample processing and a reduced number of isolated CSCs [56], which limits clinical and research application of this method [105]. Lastly, no universal marker exists for the determination of different CSCs, and the expression of the markers is highly affected by the microenvironment and cell culture conditions [105,106].

### 3.2. Side Population Assay

An important and unique characteristic of stem cells is that they usually express high levels of ATP-binding cassette (ABC) transporter protein family members, which can use ATP to pump various compounds, including drugs, out of cells [107,108,109]. High expression of these ABC transporter proteins in CSCs enables the identification of these cells as a “side population” (SP), but is also recognized as a main mechanism for CSC-mediated drug resistance [107,110,111,112]. The Hoechst SP method is commonly used to isolate stem cells by using FACS techniques [113,114,115,116,117]. SP cells are characterized by their capacity to efflux fluorescent DNA-binding dye Hoechst 33342 or Rhodamine 123 by cell membrane proteins, including the ABC family of transporters and multidrug resistance (MDR) proteins [109,118,119]. SP cells are thought to represent one of the putative CSC populations and have been identified in a diverse array of normal tissues, solid tumors, and cancer cell lines [120]. SP cells have been identified in both mouse and human mammary gland tissues [121,122], as well as in BC cell lines [110,115,116] and in BC tumors [123]. These cells are shown to display CSC properties, such as increased capability of self-renewal and tumorigenicity, when transplanted into immunocompromised mice [120]. Purified SP cells from the MCF7 breast carcinoma cell line had an increased expression of the “stemness genes” Notch 1 and β-catenin [115], featured high mammosphere formation [124], and were more radioresistant [125] and chemoresistant to agents including mitoxantrone and carboplatin than the non-SP cell population [126,127,128].

The elevated expression of ABC family transporters in stem cells is considered a crucial protective mechanism against cytotoxic substances, including drugs [129]. The main members of this family, including ABCB1 (multidrug resistance protein 1, MDR1), ABCC1, ABCF2, ABCB2, ABCC7, ABCG2, and ABAC5, are found upregulated in the SP analysis in different tumor types [111]. Among them, ABCG2 is the most important ABC transporter family member that is considered as the molecular determinant of the SP phenotype [109]. It is widely expressed in stem cell populations of various tissues as a conserved feature of stem cells from a wide variety of sources [107,115]. The elevated expression of ABCG2 has been detected in various solid tumors, including breast cancer [123,130]. ABCG2 has been found to be responsible for Hoechst 33342 dye efflux pattern and confers the SP phenotype both in humans and mice [131,132,133,134]. Vital dyes are effectively eliminated from ABCG2 and other ABC transporter protein-expressing cells. In the flow cytometric analysis, SP cells usually form a distinct small cell population (typically ~0.1%) showing little or no fluorescence with the Hoechst 33342 dye (Figure 3). Hoechst 33342 is toxic to cells at high concentrations and this is exacerbated by exposure to UV light [56]. Hoechst concentration, staining time, and staining temperature therefore need to be optimized to minimize cell toxicity [111]. A powerful and expensive 375 nm UV laser is essential to obtain clear Hoechst 33342 fluorescence signals [111]. The SP analysis and cell isolation can be performed as described by Goodell et al. [118] with modifications when necessary. A critical point in SP analysis is the way in which ABC transporter inhibitors like verapamil are used in the control samples so that the Hoechst dye can be retained. The following is the protocol used by Nahanishi et al. in isolating SP cells from breast cancer tissues and cell lines [123]. The disassociated breast cancer cells (1 × 10^6^ cells/mL) were incubated in a Dulbecco’s modified Eagle’s medium (DMEM) containing 5% fetal bovine serum (FBS), 10 mm HEPES (4-(2-hydroxyethyl)-1-piperazineethanesulfonic acid), and 5 μg/mL Hoechst 33342 for 90 min at 37 °C with or without 1 μM BCRP (breast cancer resistant protein) inhibitor Ko143 or 50 μM ABC transporter inhibitor verapamil. Samples were then analyzed by flow cytometry using a FACS Vantage SE cell sorter. To yield sufficient SP cells for in vitro and in vivo experiments, at least 10^7^ cells were recommended to be used for fluorescence-disassociated activated cell sorting [123].

Compared to other stem cell isolation methods, such as using specific cell markers, the SP assay is easier and reliable in characterization of the cell populations on viable cells [135], and the stem cells can be obtained from different tissues as long as the cells show high expression of ABC transporters [56,136]. In addition, it has a higher resolution than the conventional immunostaining assay within heterogeneous samples that uses antibodies against ABC transporters to detect the small number of SP cells (<0.5% of the total cell population) [56]. The main drawback of this assay is that there is no uniform SP staining protocol accounting for the variability of individual cell line or tissue sample properties in terms of cell numbers, dye concentration, and incubation time, which makes this method unspecified for the SP in various tissues and tumors [56].

### 3.3. Aldehyde Dehydrogenase Assay

ALDH1 catalyzes the conversion of retinol to retinoic acid in both normal and malignant stem cells, a role considered important for stem cell differentiation that leads to the normal development and homeostasis of different organs [137]. Fluorescent ALDEFLUOR assay has been used to characterize CSCs/TICs in different types of cancer, including lung cancer, prostate cancer, breast cancer, bladder cancer, liver cancer, ovarian cancer, malignant melanoma, etc., where high ALDH1 enzyme activity corresponds to CSC/TIC markers (Figure 3) [138]. ALDH1A1 and A3 are thought to be selectively expressed in BCSCs among the six ALDH1 family members and are responsible for the ALDH activity [139]; however, only the ALDH1A1 activity level was found to correlate with poorer overall survival based on the gene expression data of 3455 BC patients [140]. ALDEFLUOR assay was initially performed by Geinster et al. on both normal and malignant breast tissues [16], and later on breast cancer cell lines by different groups [67,86]. Cells with ALDH1 activity can be detected using a visible light-excitable fluorochrome, Bodipy™-aminoacetaldehyde (BAAA), which is uncharged and can freely cross the plasma membrane of intact and viable cells. Intracellular ALDH can convert BAAA into the negatively charged fluorescent product Bodipy^TM^-aminoacetate (BAA^−^) that becomes trapped in cells, because the exclusion through ABC transporter proteins is blocked by inhibitors (verapamil) added into the assay system, causing the cell to become highly fluorescent [111]. Only viable cells with an intact cell membrane can retain BAA^−^, so only viable cells can be detected and isolated using this assay [141]. As a negative control for all experiments, an equal aliquot of ALDEFLUOR-stained cells needs to be immediately quenched with ALDH inhibitor diethylaminobenzaldehyde (DEAB) [86].

The ALDEFLUOR assay can be performed as described previously [16,67]. Briefly, freshly dissociated cells from breast cancer tissues or cell lines will be incubated with the ALDEFLUOR assay buffer containing ALDH substrate BAAA (at a concentration of 1 µmol/L per 1 × 10^6^ cells) at 37 °C for 40 min in the incubator to allow substrate conversion. A negative control sample with an equal aliquot of cells under identical conditions will be immediately treated with 50 mmol/L of ALDH inhibitor DEAB after incubation. Cells with cellular fluorescence can be detected with the green fluorescence channel (FL1, 527/30 nm band-pass filter) on a flow cytometer and compared with the cells treated with ALDH inhibitor DEAB (Figure 3) [111]. The sorting gates will be established using cells that are stained with propidium iodide (PI) only for cell viability. The sorted ALDH-positive and -negative fractions can be re-evaluated for purity by flow cytometry with FACS. ALDH^+^ and ALDH^−^-sorted cell populations can then be cultured in the medium used for in vivo and in vitro experiments, such as testing the existence of stemness markers and sphere formation assay.

### 3.4. Spheroid Formation Assay

Another feature of CSCs is that they are able to create multicellular three-dimensional (3D) spheres when grown in non-adherent serum-free conditions. These spherical structures are characterized by well-rounded morphology, microsize, capacity to persist as free-floating cultures, and the presence of cancer cells [56]. Sphere formation assay has become the gold standard widely used for retrospective isolation of CSCs as well as for testing cell stemness properties. Initially, in 1992, Reynolds and Weiss developed the sphere culture method with cells isolated from an adult mammalian brain [142]. In this study, dissociated cells isolated from the central nervous system (CNS) were able to form spherical colonies and generate neurons and astrocytes during culturing. Since then, many studies have confirmed that under serum-free conditions, the CSC/TIC population can be enriched in the presence of some mitogens, such as the epithelial growth factor (EGF) and the basic fibroblast growth factor (bFGF) that support CSC growth under non-adherent conditions [142,143,144,145,146]. In this culture, the immature or undifferentiated cells grow slowly and form non-adherent clusters called tumor spheres, whereas non-malignant cells or differentiated cells die [105]. Ponti et al. found that BCSCs with the CD44^+^/CD24^−/low^ phenotype formed spheres and were enriched under low-adherent conditions in vitro [57]. ALDH1^+^ cells isolated from both normal human mammary glands (mammoshpere-initiating cells) and BC cell lines were also able to form tumorspheres [16,67].

To isolate or test the stem/progenitor cell properties of cells in vitro, the following protocol based on what Ponti et al. reported is widely used [57]. Viable single cells isolated from breast cancer patient tissues or cell line cultures were plated at 1000 cells/mL onto 60mm Petri dishes with a serum-free DMEM/F12 medium supplemented with 10 ng/mL bFGF, 20 ng/mL EGF, and the B27 supplement (1:50). Non-adherent spheroid cells, named sphere or mammosphere cells, grown in these conditions were collected and enzymatically dissociated every 3 days by incubation in a trypsin–EDTA (Ethylenediaminetetraacetic acid) solution (0.1% trypsin and 1 mM EDTA) for 2 min at 37 °C. After dissociation, 100 cells per well were plated in 96-well culture dishes in 200 µL of growth medium as described above to produce clonal spheres. 25 µL of medium per well were added every 2 days to maintain the medium level [57]. These cells were further tested for putative stem cell markers such as CD44, CD24, MUC1, and CD10 using flow cytometric analysis [57]. Cell density is the most important factor for a sphere-forming assay and should be determined based on the purpose of the individual sphere assay. To enrich for CSCs or isolate CSCs, normal cell suspensions will be plated on a Petri dish in a serum-free medium. To characterize and define the stem cell potential, i.e., self-renewal and differentiation of a newly identified population in vitro, cells should be plated as a single cell per well to ensure clonality [147].

The advantage of this assay is that it is easier than using cell surface markers or sorting them by SP to isolate cells [148]. There is also potential to identify novel CSC types with unknown cell surface markers due to the cell heterogeneity within the spheres. However, there are some disadvantages. An important drawback of this method is only a small population of cells having the self-renewal ability due to the sphere cell heterogeneity [57,149]. Other disadvantages include cell heterogeneity, differentiation potential bias, the number of passages, appropriate media and techniques needed for different types of cells [147,148], which sometimes result in conflicting results from different groups. In addition, as sphere-forming assays predominantly detect cells that are either poised for proliferation in vivo or are already actively dividing, it may not be feasible to detect quiescent stem cells in a short period of time [147]. Furthermore, not only BCSCs, but also other progenitor cells can form spheres using the sphere formation assay [150], which may lead to the overestimation of stem cell percentages in the tested cell population. Moreover, most of the currently used protocols for 3D culturing of tumor spheroids in a non-adherent serum-free suspension display several limitations and challenges pertaining to the efficient assessment of the number and size of cultured spheres, as they are mobile and can merge with each other [151]. Organoids are 3D cell cultures grown in vitro from stem cells that recapitulate the key features of both the development and performance of a native organ [152]. The development of breast cancer organoids can reproduce many of the key features of human breast cancer, thereby providing a new platform for studying BCSC properties such as self-renewal and differentiation, mimicking their in vivo counterparts.

### 3.5. Isolation and Identification Based on the Combination of Different Methods

The above-described CSC isolation methods are based on CSC properties: positive for CSC markers, part of a side population, capable of forming mammospheres, and capability to form new heterogeneous tumors in mice. However, none of these methods is exclusively used for isolation of CSCs from solid tumors as each has its limitations [153]. For example, it was reported that SP analysis alone was not able to define a CSC phenotype in glioblastoma multiforme [154] or define the CD44^+^CD24^−^ cells in breast cancer [58]. Another example is that not all stem cells show high ALDH1 expression evidenced by the fact of only partial overlapping between CD44^+^/CD24^−/low^ and ALDH^+^ BCSCs [15]. Therefore, studies have been reported to isolate CSCs in combining multiple surface markers and stem cell properties, such as combining SP and ALDH1 analysis or combining SP or ALDH1 analysis with cell surface markers [86,155,156]. The number of phenotype markers used in this kind of combination assays depends on the equipment used for flow cytometry and the availability of appropriate antibody conjugates [111]. In addition, the isolation assay with combined procedures needs to take into account the sequence of the different protocols, because some staining procedures or chemicals used are not compatible. One example is that verapamil can be used in both the ALDEFLUOR assay and SP assays, although with different purposes. In the ALDH1 assay, verapamil is used as a channel inhibitor to prevent the active efflux of ALDH1 substrate BAAA, whereas in the SP assay, it acts as an inhibitor in the control group. Thus, Pierre–Louis et al. proposed to stain the SP cells first and then perform the ALDH1 staining with the analysis of additional phenotype markers as the final step [156]. Using this combinatory assay method, Pierre–Louis et al. reported that the co-expression of SP and ALDH markers refines the Lin^−^CD34^+^CD38^−^ hematopoietic compartment and identified an SP/ALDH^Bright^ cell subset enriched with quiescent primitive hematopoietic stem cells [156]. Similarly, Pearce and Bonnet performed simultaneous phenotyping with Hoechst exclusion and ALDH labeling and found that SP techniques identified cells that overlap with the ALDH^+^ cell population with the capability of long-term repopulation [155]. By combining the ALDH activity assay and cell surface markers, ALDH^hi^CD44^+^CD24^−^ and ALDH^hi^CD44^+^CD133^+^ cells were found to have an enhanced malignant and metastatic ability in comparison with ALDH^low^CD44^+^CD24^−^ and ALDH^low^CD44^+^CD133^+^ counterparts [16,86,157].

## 4. Signaling Pathways and Molecules in the Regulation of BCSCs

The major signaling pathways in regulating BCSC properties, i.e., stemness, self-renewal, metastasis, and therapeutic resistance, include Notch, Wnt/β-catenin, hedgehog (Hh), and Hippo signaling pathways [158,159]. It has been noted that pathway deregulations such as genetic mutations encoding proteins involved in these critical pathways in normal stem cells lead to the transformation of these cells into BCSCs and eventually uncontrolled cell proliferation to form tumors. Besides these signaling pathways, non-coding RNAs (ncRNAs), mainly microRNAs (miRNAs) and long non-coding RNAs (lincRNAs) are also important regulators in BCSCs.

### 4.1. Major Signaling Pathways That Regulate BCSCs

The Notch signaling pathway is a highly conserved molecular signaling pathway that plays a vital role in self-renewal, stem cell maintenance, and cellular differentiation during the development stage of cells and serves as a key signaling cascade involved in the maintenance of the BCSC phenotype [160]. The Notch family consists of four receptors, Notch 1–4, and these receptors are known to bind to five different ligands in adjacent cells: jagged proteins (JAG1 and JAG2) and delta-like proteins (DLL1, DLL3, and DLL4) to activate Notch signaling [161]. In the mouse mammary gland, Notch signaling was shown to regulate the expansion of stem cells and differentiation into luminal progenitor cells [162]. Upregulated expression/activity of Notch was found in BCSCs and this activity was linked to tumor-initiating properties and CSC-like invasive features [163,164]. The tumor growth was arrested and both the CD44^+^/CD24^−/low^ and ALDH^+^ BCSC populations were decreased in the xenograft model with the inhibition of γ-secretase, which prevents the formation of the active Notch intracellular domain (NCID) [165,166,167]. In addition, upregulation of the Notch signaling by overexpression of Notch ligand DLL1 promotes proliferation, migration, angiogenesis, and the CSC phenotype in ERα+ BC cells [168]. Recent studies have shown that in BCSCs, jagged1 protein and cartilage oligomeric matrix protein (COMP) are involved in the activation of Notch signaling through the activation of Notch 1 and Notch 3, which contribute to the maintenance of BCSCs properties [169,170].

The Wnt/β-catenin signaling pathway plays a crucial role in regulating stem cell division and self-renewal. Previous studies have shown that oncogenic activation of the Wnt/β-catenin signaling enrich mammary stem and progenitor cells [171,172], as well as increased human mammosphere formation [173]. Recently, Wnt signaling has been found to be important in maintaining the activity of ALDH-positive BCSCs in TNBC cells [174]. Activated Wnt/β-catenin signaling was found to be involved in BC chemoresistance and radioresistance [175], while blockage of the Wnt/β-catenin signaling suppresses breast cancer metastasis through the inhibition of the CSC-like phenotype [176]. Interestingly, it was found that overexpression of programmed death-ligand 1 (PD-L1) in BCSCs mediates the contribution of Wnt signaling into the stemness phenotype of CSCs [177], indicating a potential of immunotherapy with anti-PD-L1 inhibitor to help eradicate BCSCs. Other recently reported molecules that are involved in the regulation of Wnt signaling pathways include KIF11 [178], Nectin 4 [179], and cytokeratin 5 [180]. Regulation of the Wnt signaling pathway by those molecules contribute to the BC metastasis and proliferation of BCSCs. Both Wnt and Notch signaling may be activated by HIF-2α overexpression that promotes the stem cell phenotype, drug resistance of BCSCs, and overexpression of BCSC markers [181].

The Hh pathway plays an important role in various cellular processes during embryonic development and is a key regulator of cell fate through the regulation of cell proliferation and differentiation [182]. It has been found to play an important role in normal and malignant breast stem cells. Overexpression of the key Hh pathway regulators, SHH, DHH, PTCH1, and GLI1, in malignant tumors was found to be associated with proliferation, migration, metastasis, and aggressiveness of BC [183,184]. The previous study showed that Indian Hh, PTCH1, SMOH, GLI1, and GLI2 are expressed in stem and progenitor cells when cultured as mammospheres, while their expression was greatly reduced when cells underwent differentiation [185]. Moveover, activated Hh signaling with higher expression of SMOH and GLI1 was noted in the BCSCs characterized as the CD44^+^/CD24^−^/Lin^−^/ALDH1^+^ phenotype compared to bulk BC cells to retain the stemness potential [186]. In addition, overexpression of Hh signaling molecules, such as SHH, PTCH1, and GLIs, is associated with angiogenesis, extracellular matrix degradation, and metastasis [184].

Another important pathway involved in CSCs is the Hippo pathway that plays an important role in organogenesis and regeneration [187]. The Hippo pathway comprises a core regulatory kinase module and a core transcriptional module. The first component includes a set of kinases MST1, MST2, LAST1 and LAST2, SAV1, MOB1A and MOB1B. The latter encompasses two closely related transcriptional paralogs TAZ and YAP [188]. Expression of TAZ and YAP was identified in different BC subtypes with various degrees, and TAZ had a significantly increased expression in BCSC-derived tumors compared to non-BCSC-derived tumors [188]. Treatment with a multi-target kinase inhibitor, dasatinib, proved able to selectively kill the CSC population in a TAZ-driven model [189]. Exposure to dasatinib led to the inhibition of anchorage-independent growth, impaired mammosphere-forming ability, as well as depleted the CD44^+^/CD24^−^ subpopulation [189]. Interaction between TAZ and the extracellular matrix was shown to be responsible for the maintenance of the BCSC pool [190]. A recent study showed that overexpression of Hippo pathway component LAST2 could reduce the breast cancer stemness induced by miR-520b upregulation [191].

### 4.2. The Role of Non-Coding RNAs in BCSCs

MiRNAs are endogenous non-coding ~20–23 nt RNAs processed from larger hairpin structures that bind to complementary sequences in the 3′-untranslated regions (UTR) of target mRNAs to regulate the expression of the target genes. Many miRNAs, including but not limited to miR-155, miR-140, miR-21, miR-22, miR-24, miR208a, miR-10b, miR-27a, miR-99a, miR-29b, miR-34, miR-221/222, miR-142, miR-520b, Let-7, and miR-30, are found to play critical roles in maintaining BCSC properties and/or drug resistance [72,161,192]. Among them, some miRNAs, such as miR-24, miR-21, miR-22, and miR-221/222, have an oncogenic function, and the increased expression of them will promote BCSC properties and increase drug resistance [72,161,192]. At the same time, some miRNAs, such as the miR-200 family, miR-128, miR-600, Let-7c, miR-30, miR-34, and miR-489, work as tumor suppressors, and the overexpression of these miRNAs can decrease the stemness of BCSCs or reverse drug resistance [192,193]. These miRNAs play important roles in regulating BCSC self-renewal, mediating tumor metastasis and drug resistance through different mechanisms. For example, several miRNAs, including Let-7, miR-146, miR-142, miR-374, miR-600, and miR-340, are found to control the BCSC phenotype through the regulation of the Wnt signaling pathway [161,192]. The miR-200 family, miR-9, and miR-34c were reported to suppress Notch signaling by targeting Notch pathway components to reduce the metastatic behavior of TNBC [192].

LincRNAs are a series of transcripts longer than 200 nucleotides that have no potential of protein coding [194], but can recruit transcription factors to regulate gene expression, or interact with miRNAs to influence the stability of mRNAs [195]. In recent years, lincRNAs, HOTAIR (HOX transcript antisense RNA) [196], ROR (regulator of reprogramming) [197], and 00617 [198] have been shown to be involved in BCSCs through the regulation of EMT signaling pathways [72]. Zheng et al. reported that LincRNA LUCAT1 forms an axis with miR-5582-3p and TCF7L2 to regulate BC stemness via the Wnt/β-catenin pathway [199]. Other lincRNAs such as NRAD1 [200], NEAT1 [201], H19 [202], FGF13-AS1 [203], SOX2OT [204], MALAT-1 [205], and ES1 [206] were also shown to be involved in stemness of BC via different mechanisms. These ncRNAs provide insights for regulatory mechanisms of stemness and are potential biomarkers and therapeutic targets for designing the BCSC-directed therapy.

### 4.3. Therapeutic Drugs Targeting BCSC Subpopulations

Numerous drugs have been generated to target the key BCSC signaling pathways of Wnt, Notch, and Hh, as well as to target the pathways and factors that modulate the activities of these pathways as reviewed in [72,207]. Some of the drugs have been tested in clinical trials. For example, γ-secretase inhibitor MK-0752 from Merck completed phase I/II clinical trials for metastatic BC, and patient biopsy samples showed a decrease in cell population with the CD44^+^/CD24^−^ and ALDH^+^ phenotype, which was the first evidence of the benefits of the BCSC-targeted therapy through targeting the Notch signaling pathway in combination with docetaxel as the systemic cytotoxic therapy [72,208]. Two monoclonal antibodies, vantictumab (OMP-18R5) and cirtuzumab (UC-961), targeting Wnt signaling pathway components Frizzled and ROR1, respectively, were tested for metastatic BC [72]. LGK-974, an inhibitor of the endogenous Wnt palmitoleoylase Porcupine (PORCN) that is required for palmitoylation of Wnt ligands Wnt5a and Wnt5b before their secretion, has been clinically trialed for BC alone or in combination with immunotherapy treatment [72,209].

## 5. Origin of BCSCs and Methods for Studying BC Origin and Lineage Development

Although the critical roles played by BCSCs in tumor initiation, progression, and resistance to therapies are established, the origin of BCSCs remains elusive. Traditional single-cell lineage tracing assay has been the gold standard to decipher the tumor origin; however, this method has limitations, as cell type identification is usually based on a limited number of markers, and the progeny from these marked cells is developmentally related [210]. Recent development of single-cell transcriptomics and single-cell genetic lineage tracing has opened a new avenue in the study of BCSC origin.

### 5.1. Origin of BCSCs and Traditional Methods for BCSC Lineage Tracing

Currently, three main hypotheses exist about the origin of BCSCs: formation from normal stem cells, mutation-induced pluripotency of progenitor cells into cancer stem cells, and dedifferentiation of adult mammary cells into stem cells through the epithelial-to-mesenchymal transition [211]. The concept of BCSCs arising from either mammary stem cells or progenitor cells seems to be more plausible based on the current knowledge of BCSCs [84,212,213]. This model postulates that within a tumor, only a small proportion of stem/progenitor cells possess the tumor-propagating potential and can reiterate tumor hierarchy; therefore, it is called a hierarchical or an CSC model. As is the case of normal stem cells, BCSCs can be isolated based on the elevated ALDH1 expression [16]. Both normal and cancer human mammary epithelial cells with increased ALDH1 activity were demonstrated to show stem/progenitor cell properties that could initiate tumors in both in vivo and in vitro experiments [16]. Studies also reported that the CD44^+^/CD24^−^ cell marker expressed on progenitor cells resembles the CD44^+^/CD24^−^/Lin^−^ phenotype found in BCSCs [84]. In addition, gene expression profiling also revealed that basal breast tumors were more similar to normal luminal progenitor cells than to any other epithelial subset, including the stem cell-enriched population [213]. Furthermore, BCSCs share highly similar properties to mammary stem cells and lightly differentiated progenitor cells with self-renewal, proliferation, and differentiation capabilities [16,38,84,212]. BCSCs form mammoshperes in vitro [57], generate tumors that recapitulate the phenotypic heterogeneity of the initial tumor [38], are involved in tumor metastasis, and are resistant to conventional therapy [2].

In spite of the above evidence, studies also suggest that differentiated mammary cells can transform to BCSCs under exposure to damaging environmental factors such as radiotherapy and chemotherapy that may lead to genetic alterations of cells, enabling them to regain stem cell-like properties through cell dedifferentiation [2,214,215,216,217]. In this model, all tumor cells are considered to have similar tumorigenic potential and the intra-tumoral clonal evolution through sequential mutations gives rise to tumor heterogeneity, which is called clonal evolution or the stochastic model. Van Keymeulen et al. [216] reported that a PIK3CA mutant in luminal cells induced both luminal and basal-like tumors, while its expression in basal cells gave rise to luminal tumors, indicating the reactivation of multipotency from differentiated mammary cells. Similarly, another study showed that under certain environmental stimuli, such as when co-inoculated with the irradiated cells, tumor cells with the stem cell-, basal-, and luminal-like phenotype were equally tumorigenic, and each tumor cell subpopulation could generate xenografts [218]. However, under standard conditions, only stem cells efficiently generated tumors when xenotransplanted into mice. This study indicates that CSC and non-CSC states are not hardwired and interconversion can happen under specific conditions [218].

### 5.2. Traditional Methods for BCSC Lineage Tracing

To define the origin of BCSCs, the current gold standard in the laboratory is the lineage tracing assay in mouse models. In this assay, distinct cell subpopulations are labeled using different cell-specific promotors, which allows tracking of a single-cell-derived clone in animals [219]. In recent years, lineage tracing has been mainly performed through tracking of genetic features followed by introduction of activating or inactivating mutations in a variety of oncogenes and tumor suppressors in the same cell type, enabling the transformed cell that forms a tumor to be traced back and identified as the cellular source of the tumor. This method relies on the introduction of reporter transgenes, such as β-galactosidase or green fluorescence protein (GFP), so that the transformed cells can be traced and visualized [220,221]. After the tumor is established, the positively marked cells can be purified out of the traced tumor and further tested to determine whether they have CSC properties through limiting dilution assay and serial transplantation assay [219]. Via lineage tracing, PROCR, a novel Wnt target in mammary epithelial cells, was found to define a rare unique subset of multipotent mouse mammary stem cells [62]. The serial transplantation assay is considered the gold standard for identifying CSCs, as it can assess CSC properties of self-renewal and multiplicity; however, it can also be used to study the origin of cancer cells. In order to determine the potential origin of BCSCs, normal cell subpopulations are sorted with FACS based on specific markers followed by genetic alterations to overexpress oncogenes or knock out tumor suppressor genes. During the subsequent xenotransplantation assays for assessing the differential tumor formation potential of different cells, cell populations positive for specific markers are considered the potential cells-of-origin if they give rise to tumors that resemble parental or patient tumors [219].

### 5.3. Single-Cell Transcriptomics in the Study of BCSC Lineages

Both single-cell lineage tracing and serial transplantation assay for cell type identification are usually based on a limited number of markers, and all the progeny is originated from the same founder cell, which may harm the accuracy and precision of cell type classification and potentially mask the variability within a subpopulation of cells that express the selected marker genes [210]. Recent advances in single-cell RNA sequencing (scRNA-seq) that captures different cell states within a developmental or differentiation trajectory can be used to recapitulate lineages, allowing for the unbiased characterization of molecular states and cell identities at unprecedented resolution in a heterogeneous tissue [210,222,223,224,225]. Gulati et al. reported that mature luminal (ML) subpopulations are downstream of less differentiated luminal progenitor (LP) cells in TNBC tumors through the scRNA-seq profiling of breast tumor epithelial cells and adjacent normal epithelial cells from TNBC patients, indicating the LP cells as the plausible origin of BCSCs [225]. Based on single-cell transcriptomes, Giraddi et al. found that fetal mammary stem cells show co-expression of factors and a metabolic gene signature resembling that of human breast cancers and metastases [226]. Nguyen et al. performed scRNA-seq analysis on FACS-sorted breast epithelial cells, and proposed a continuous lineage hierarchy connecting the basal lineage to the luminal progenitor and mature luminal cells via bipotent mammary stem cells (MaSCs) [223]. This result is in agreement with previous findings suggesting that MaSCs are bipotent and can contribute to both the basal and luminal cell lineages [227]. These studies indicate mammary stem/progenitor cells as the origin of BCSCs.

### 5.4. Combination of Single-Cell RNA-seq and Single-Cell Genetic Barcode Tracing in the Study of the BCSC Origin

**Figure 4:**
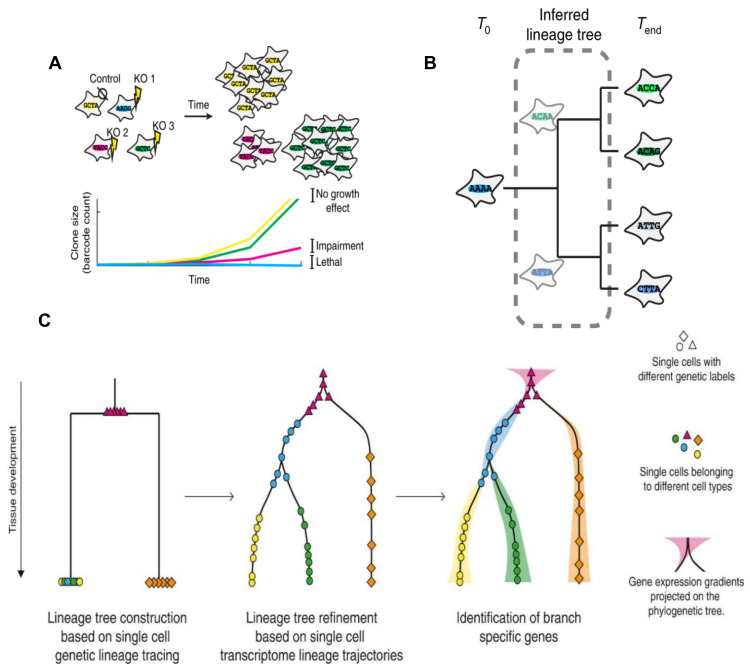
Application of cellular barcoding and lineage tree reconstruction based on single-cell lineage tracing and transcriptomics. (**A**) Cellular barcoding can be applied to cell fate mapping studies, such as to count the number of dividing stem cells in the stem cell niche to study the heterogeneity and clonality in breast tumors based on the number of labeled expanded lineages. (**B**) Evolving barcodes, such as those generated by CRISPR-Cas9 barcoding, allow the reconstruction of cell lineages in a single experiment through retrospective inference of cellular relationships on the basis of barcode similarity. Lineage relationships between cells at the experimental endpoint can be inferred from barcode similarity. (**C**) Lineage tree reconstruction based on the combination of single-cell genetic lineage tracing and single-cell transcriptomics. The first step is to construct a phylogenetic tree based on the genetic labels identified in single cells such as added DNA barcoding or CRSPR-Cas9 barcoding. Transcriptomics-based lineage reconstruction algorithms can then be used to refine this tree. Finally, gene expression gradients or patterns can be projected onto the phylogenetic tree to identify gene expression dynamics throughout the system. This figure is adopted from [210,228].

The scRNA-seq made it possible to get a snapshot of the transcriptome of thousands of single cells and allow for detailed cell type classification; however, the challenge remains because of the difficulty in tracking individual cells over space and time with similar high throughput technology [228]. In recent years, other more advanced lineage tracing methods were developed to track a larger number of clones in complex tissues using nucleotide sequences as lineage barcodes, including viral barcoding to induce cell-specific DNA barcodes [229], Cre-Lox system-mediated recombination-based barcoding that can control cell activation at any time [230], and CRISPR-Cas9 genome editing-based lineage tracing [231,232]. These genetic lineage tracing will be able to reveal the clonal relationships of the cells, help to study heterogeneity and clonality in cancer through cell fate mapping, or retrospectively study lineage development based on evolving barcodes to reconstruct the lineage tree from a single experiment (Figure 4A,B). The integration of single-cell lineage tracing and transcriptomics will allow lineage reconstruction based on genetically heritable marks, which can be further refined based on the transcriptome-derived differentiation trajectories and the assessment of gene expression changes over the developmental stages [210,228,233] (Figure 4C).

In the past few years, several publications showcased the true potential of combining single-cell transcriptomics with single-cell level genetic lineage tracing in providing information on the relationships between cells for lineage reconstruction along with detailed phenotypic information [210,222,233,234,235,236,237,238]. For example, the study of hematopoietic stem cells with both single-cell transcriptomics [239] and single-cell lineage tracing [240] have shown that even within the stem cell compartment cells develop with a bias towards a certain fate instead of with unlimited potential, while the fate of progenitor cells is not strict (predetermined) as previously thought [210]. This indicates a continuous differentiation process of hematopoietic cells, which is in contrast to the classical lineage tree theory where differentiation occurs as discrete steps with stem cells and progenitor cells being completely separated [210,241]. However, these lineage tracing studies require genome editing to introduce traceable elements, limiting the use of these techniques only to the model organism or to in vitro systems. Future studies combining existing epigenetic marks and single-cell transcriptomics will make it possible to use human samples for studying human development and diseases such as cancer, which may eventually solve the mystery of the origin of BCSCs.

## 6. Conclusions

Increasing evidence has proved the existence of BCSCs and their function during tumor initiation, progression, metastasis, drug resistance, and tumor recurrence. In the past decade, the study of BCSCs has significantly advanced our knowledge in the identification, isolation, and characterization of this specific cell population. The understanding of the signaling pathways and molecules involved in the maintenance of BCSC characteristics and tumor stemness has enabled the development of targeted therapy for BCSCs. The current therapies targeting the BCSC subpopulations mainly include the molecules inhibiting the quiescent state of BCSCs, resensitizing BCSCs from radiation and chemotherapy resistance, and targeting the BCSC signaling pathways and the factors that maintain BCSC properties. Treatment regimens of combining BCSC-targeted therapy and systemic therapy are recommended because of the distinct properties of BCSCs from the rest of tumor cells, such as the quiescent nature of BCSCs which makes them resistant to conventional chemotherapy that normally targets rapidly dividing cells. Despite the advanced understanding of multiple aspects of BCSCs, the origin of BCSCs is still elusive. The proportion of BCSCs and relative abundance of different subtypes of BCSCs at different stages of tumor progression as well as how they compete or collaborate during this process still need to be evaluated. Recent advancement of the integration of single-cell genetic/epigenetic lineage tracing and single-cell transcriptomics technology along with the novel computational algorithms of lineage reconstruction and differentiation trajectories has opened a new avenue in deciphering the lineage development of complex tissues or organisms, which may eventually lead to the identification of the BCSC origin.
